# Correction: Yu et al. Characterization of Two Novel Single-Stranded RNA Viruses from *Agroathelia rolfsii*, the Causal Agent of Peanut Stem Rot. *Viruses* 2024, *16*, 854

**DOI:** 10.3390/v17030316

**Published:** 2025-02-26

**Authors:** Dongyang Yu, Qianqian Wang, Wanduo Song, Yanping Kang, Yong Lei, Zhihui Wang, Yuning Chen, Dongxin Huai, Xin Wang, Boshou Liao, Liying Yan

**Affiliations:** Oil Crops Research Institute, Chinese Academy of Agricultural Sciences/Key Laboratory of Biology and Genetic Improvement of Oil Crops, Ministry of Agricultural and Rural Affairs, Wuhan 430062, China; yudongyang0730@aliyun.com (D.Y.); wangqianqian01@caas.cn (Q.W.); songwanduo@caas.cn (W.S.); kangyanping@caas.cn (Y.K.); leiyong@caas.cn (Y.L.); wangzhihui@caas.cn (Z.W.); chenyuning@caas.cn (Y.C.); dxhuai@163.com (D.H.); wangxin456_2000@163.com (X.W.)

## Error in Table

In the original publication [1], there was a mistake in the content of Table 3 as published. The lack of GP3-1 as a control in the published article also resulted in errors in the data of the viral amount. The corrected content of Table 3 appears below. The authors state that the scientific conclusions are unaffected. This correction was approved by the Academic Editor. The original publication has also been updated.

## Figures and Tables

**Table 3:** Correlation analysis of viral amount and diameter of lesions on the leaves.

Mycovirus	Strain	Virus Amount	Lesion Diameter (cm)	Significance
	GP3-1	2.568 ± 0.046	0.7 ± 0.03	−0.981 **
ArMV1	BL1-1	1.014 ± 0.005	1.4 ± 0.06	
	BG5	1.638 ± 0.012	1.1 ± 0.05	
	BG10	2.405 ± 0.032	0.8 ± 0.04	
	GP3-1	0.465 ± 0.024	0.7 ± 0.03	−0.967 **
ArMV2	BL1-1	0.299 ± 0.0179	1.4 ± 0.06	
	BG5	0.342 ± 0.017	1.1 ± 0.05	
	BG10	0.480 ± 0.018	0.8 ± 0.04	

Note: ** denotes *p* < 0.01.
