# Supplementary material for: Characterization of Two Novel Single-Stranded RNA Viruses from Agroathelia rolfsii, the Causal Agent of Peanut Stem Rot
Source: Viruses. 2024 May 27;16(6):854. doi: 10.3390/v16060854 (PMC11209298; doi:10.3390/v16060854)
Supplement: Supplementary file 1 [file viruses-16-00854-s001.zip › viruses-2976492-supplementary.pdf]

Supplementary Materials

Table S1. The information of primers used in this study.

Primer Name	Primer Sequence
1-F1	ACGCAACTATATTTTCAAAGT
1-R1	GCATTAAGAGCAGCTACTTGC
1-F2	TCCACTATTCAGTGGGGCAA
1-R2	GCTCTTCTAGAGCTTCAGG
1-F3	AGAATTCGGGCCTTGTTAG
1-R3	CACAGTATCATGGGCTAGGG
2-F1	GTCTGTCATGGCCTTTAGA
2-R1	ATAGTCACTCAGTAGCGGTC
2-F2	CTAATCCACTAACGGTGGTG
2-R2	CCTCACCAGAAGGTGAAAG
2-F3	GACTGTCCGATATTCTGTCG
2-R3	CAGGTGTTGGAGCAATCCAA
Ra2-R1	ATCATGGCAGCTATGTTGCT
Ra2-F1	ATACGATTTTCGTTCCATCTGA
Ra2-R1	TCTAAAGGCCATGACAGACA
Ra2-F1	TTACAGACTCTATGAGTCTCGC

Table S2. The information of RdRp domain of viruses selected for multiple sequence alignment analysis and phylogenetic analysis in this study.

Virus Name	Accession	RdRp(aa)
Agaricus bisporus mitovirus 1	YP_010799232.1	956
Alternaria alternata mitovirus 1	YP_010799907.1	850
Azolla filiculoides mitovirus 1	YP_010799907.1	850
Beta vulgaris mitovirus 1	YP_010799341.1	793
Cannabis sativa mitovirus 1	WJJ80270.1	762
Chenopodium quinoa mitovirus 1	QLK97559.1	766
Colletotrichum falcatum mitovirus 1	AZT88621.1	703
Colletotrichum fructicola mitovirus 1	BBN51032.1	709
Cronartium ribicola mitovirus 1	YP_009259369.1	800
Diaporthe gulyae mitovirus 1	WNM95029.1	724
Diaporthe helianthi mitovirus 1	WNM95050.1	706
Diaporthe rudis mitovirus 1	YP_010800592.1	701
Entomophthora muscae mitovirus 1	YP_010799924.1	757
Erigeron breviscapus mitovirus 1	DAB41748.1	780
Erysiphe necator associated mitovirus 1	QHD64812.1	721
Erysiphe necator associated mitovirus 1	QHD64812.1	721
Fomitiporia mediterranea mitovirus 1	QDB74991.1	666
Fusarium boothii mitovirus 1	YP_010799254.1	823

Fusarium sambucinum mitovirus 1	BCP96872.1	783
Geopora sumneriana mitovirus 1	YP_010799955.1	827
Humulus lupulus mitovirus 1	YP_010798879.1	763
Ophiostoma mitovirus 4	NP_660179.1	783
Ophiostoma mitovirus 7	YP_010799133.1	720
Oxybasis rubra mitovirus 1	YP_010798876.1	763
Petunia exserta mitovirus 1	YP_010798875.1	750
Rhizoctonia mitovirus 1	YP_009551966.1	826
Rhizoctonia mitovirus 1 RS002	YP_010799131.1	826
Rhizoctonia solani mitovirus 115	WAY16594.1	939
Rhizoctonia solani mitovirus 23	YP_010799583.1	818
Rhizoctonia solani mitovirus 25	YP_010799572.1	861
Rhizoctonia solani mitovirus 3	YP_010800186.1	840
Rhizoctonia solani mitovirus 30	YP_010799575.1	818
Rhizophagus diaphanum mitovirus 1	YP_009553678.1	812
Rhizophagus irregularis mitovirus 1	YP_009552077.1	811
Sclerotinia homoeocarpa mitovirus	YP_010798871.1	720
Sclerotinia sclerotiorum mitovirus 11	AHF48627.1	683
Sclerotinia sclerotiorum mitovirus 3	YP_010799120.1	712
Thielaviopsis basicola mitovirus	YP_002822229.1	705
Tuber excavatum mitovirus	YP_010799117.1	797

Table S3. The information of isolate selected for determination of mitocovirus in other *A. rolfsii* strains in this study.

**Isolate**	**Strain source (Province)**
BS1	Chongqing
BSH1	Fujian
CHJ1	Jiangxi
FJT	Jilin
GZH1	Guizhou
HN1	Hainan
KL1	Guangdong
JZ1	Shandong
SX1	Anhui
TSH1	Liaoning
XJ1	Hebei
YD1	Sichuan
YL1	Huibei
ZY2	Henan

Table S4. Information of two viral contigs in strain GP3-1.

Cintug Number	Length (bp)	Best Match (Blastp)	Accession	Identity	Query Cover	Family
contig1	3514	Rhizoctonia solani mitovirus 115	WAY16594.1	36.27%	78%	Mitovirus
contig2	3410	Rhizoctonia solani mitovirus 115	WAY16594.1	35.12%	83%	Mitovirus

Table S5. The information of mitovirus in other hosts selected for the multiple comparison diagrams of conserved domains in this study.

Virus Name	Accession	Host
Rhizoctonia solani mitovirus 115	WAY16594.1	Rhizoctonia solani
Sclerotinia sclerotiorum mitovirus 11	AHF48627.1	Sclerotinia sclerotiorum
Rhizophagus diaphanum mitovirus 1	YP_009553678.1	Rhizophagus diaphanum
Fusarium sambucinum mitovirus 1	BCP96872.1	Fusarium sambucinum
Agaricus bisporus mitovirus 1	YP_010799232.1	Agaricus bisporus
Ocimum basilicum RNA virus 2	YP_009408146.1	Ocimum basilicum

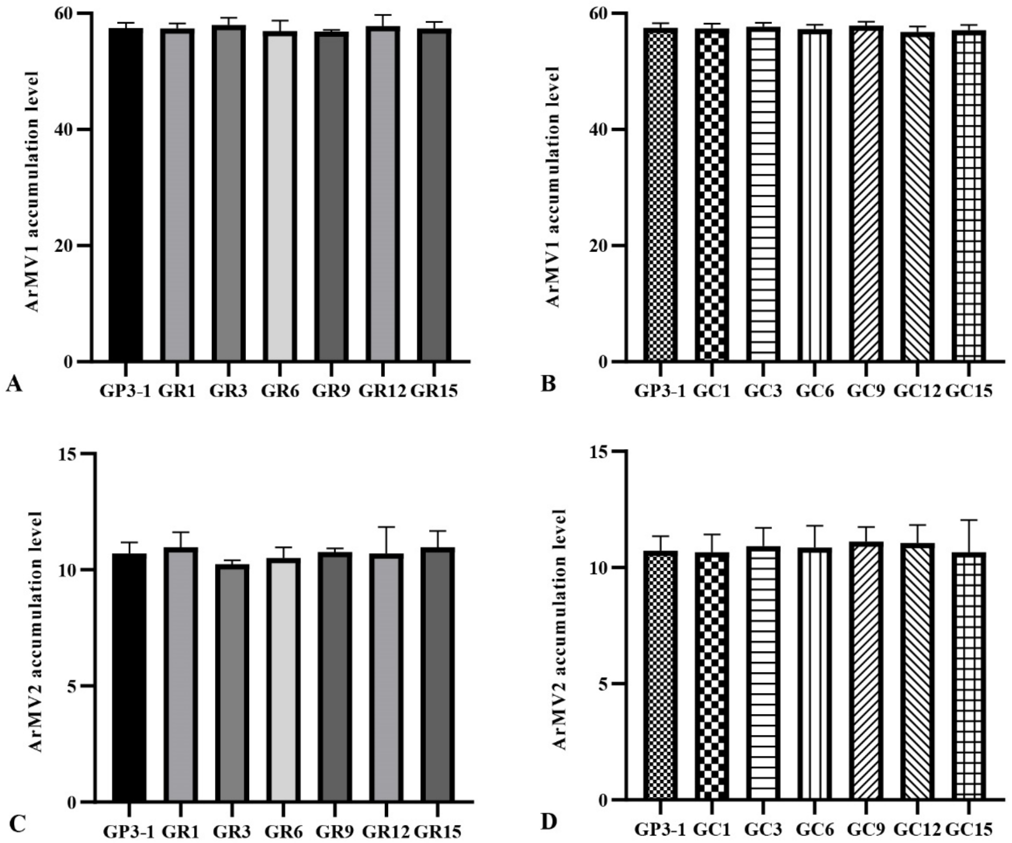

Figure S1. The difference of ArMV1 and ArMV2 accumulation level in virus-free GP3-1. (A) ArMV1 accumulation level of GP3-1 treated with ribavirin. (B) ArMV1 accumulation level of GP3-1 treated with cycloheximide. (C) ArMV2 accumulation level of GP3-1 treated with

ribavirin. (D) ArMV2 accumulation level of GP3-1 treated with cycloheximide. GP means GP3-1 treated with ribavirin; GC means GP3-1 treated with cycloheximide; Number means the times GP3-1 treated with ribavirin or cycloheximide.
